# Effects of renin‐angiotensin blockade and APOL1 on kidney function in sickle cell disease

**DOI:** 10.1002/jha2.259

**Published:** 2021-07-08

**Authors:** Jin Han, Andrew Srisuwananukorn, Binal N. Shah, Robert E. Molokie, James P. Lash, Victor R. Gordeuk, Santosh L. Saraf

**Affiliations:** ^1^ Department of Pharmacy Practice, College of Pharmacy University of Illinois at Chicago Chicago Illinois USA; ^2^ Division of Hematology and Oncology, Department of Medicine University of Illinois at Chicago Chicago Illinois USA; ^3^ Department of Medicine Jesse Brown VA Medical Center Chicago Illinois USA; ^4^ Division of Nephrology, Department of Medicine University of Illinois at Chicago Chicago Illinois USA

**Keywords:** angiotensin converting enzyme inhibitors, angiotensin receptor blockers, APOL1, sickle cell disease

Kidney disease is a common complication that leads to increased morbidity and early mortality in patients with sickle cell disease (SCD).[1] Treatments for SCD‐related kidney disease have been adopted from therapies used to treat other causes of kidney disease (angiotensin converting enzyme‐inhibitor or angiotensin receptor blocker [ACEi/ARB]), although their safety and effects on kidney function are not clear.[2] Homozygous or compound heterozygous inheritance of the *APOL1* G1 and G2 kidney risk variants increases the risk for chronic kidney disease (CKD) approximately seven‐fold in SCD.[3] The potential benefit for ACEi to reduce the risk of kidney disease progression in African Americans with the *APOL1* kidney risk variants has been observed in the African American Study of Kidney Disease and Hypertension (AASK) cohort.[4] In contrast, ACEi/ARB therapy did not reduce the risk for progression to end‐stage kidney disease in people with HIV‐related kidney disease and the *APOL1* kidney risk variants.[5] The effects of ACEi/ARB on kidney function in those that have inherited SCD and the *APOL1* kidney risk variants are not well understood. We hypothesized that co‐inheritance of the *APOL1* kidney risk variants may reduce the benefit of ACEi/ARB on the reduction of albuminuria in SCD.

We identified 47 SCD patients enrolled in a prospective observational registry who were initiated on an ACE‐inhibitor or ARB between 2011 and 2019. The study was approved by the University of Illinois at Chicago Institutional Review Board, and all patients provided written informed consent prior to blood sampling and clinical data collection. Patients were considered for this analysis if they had 6 months of kidney function values pre‐therapy and remained on therapy for 6 months or longer. The decision to start ACEi/ARB was made by the patients’ treating physicians. Genotyping for the *APOL1* G1 and G2 variants was conducted by Taqman genotyping assays (Applied Biostystems, Foster City, CA, USA) as previously described.[3] Changes in the estimated glomerular filtration rate (eGFR), as calculated by the CKD‐Epidemiology formula,[6] and urine albumin‐to‐creatinine ratio (ACR) concentration were compared prior to and after starting therapy using a mixed effects model (919 observations for eGFR and 310 observations for urine ACR). Sequential adjustments for covariates were performed as follows: Model 1 = age, sex, SCD genotype; Model 2 = added *APOL1* high risk (defined as homozygosity or compound heterozygosity for G1 and G2).

In 47 SCD patients started on ACEi/ARB, the median age was 43 years (interquartile range [IQR], 33–54 years), 43% were male, 87% were Hb SS/Sβ^0^‐thalassemia genotype, 17% were on chronic non‐steroidal anti‐inflammatory drugs, 49% were on HU, and 13% had high risk *APOL1*. The baseline median eGFR was 118 (IQR, 87–139) ml/min/1.73m^2^, and urine ACR was 263 (IQR, 63–640) mg/g creatinine. The ACEi/ARB therapy was lisinopril in 31 (66%), losartan in 14 (30%), and valsartan in two (4%) patients. The median duration of therapy was 20 months (IQR, 13–46 months), and 34 of 47 (72%) patients remained on ACEi/ARB therapy at the last time of follow‐up. Reasons for discontinuation included adverse effects (acute worsening of kidney function: *n* = 5, hyperkalemia: *n* = 3, hypotension: *n* = 1), pregnancy (*n* = 2), or patient declining to continue ACEi/ARB therapy (*n* = 2). The SCD patients that co‐inherited *APOL1* high risk had a higher frequency of adverse effects requiring discontinuation of ACEi/ARB therapy (high risk: 3/6 vs. normal risk: 6/41; *p* = 0.03). The adverse effects that led to discontinuation in SCD patients with *APOL1* high risk included acute kidney injury (*n* = 2) and hypotension (*n* = 1).

In the unadjusted and adjusted models, there was no observed difference in the change in eGFR pre‐ versus during ACEi/ARB therapy. The urine ACR improved from pre‐therapy (6‐month ß [95% confidence interval, CI]: −0.1 (−0.6 to +0.5) mg/g creatinine) to during therapy (6‐month ß (95% CI): −0.5 (−0.7 to −0.2) mg/g creatinine) (*p* = 0.01) (**Table** **1**). High‐risk *APOL1* status was an independent predictor for an increase in urine ACR during the study period (6‐month ß [95% CI]: +1.35 [+0.2 to +2.3] mg/g creatinine; *p* = 0.02), after adjusting for age, sex, SCD genotype, and ACEi/ARB use. A trend for an improvement with ACEi/ARB therapy was observed in the fully adjusted model that included *APOL1* high risk status (**Table** **1**).

**TABLE 1:** Change in eGFR and urine ACR with ACEi/ARBS as per 6‐month increments

Measure of kidney function	Model	Pre‐therapy	During therapy	*p* value
**eGFR**	Unadjusted	+12.4 (−13.9 to +38.8)	+ 11.6 (+0.5 to + 22.7)	0.9
	Model 1	+9.6 (−14.6 to +33.7)	+5.6 (−4.5 to 15.8)	0.6
	Model 2	+7.7 (−11.8 to +27.4)	+6.4 (−2.6 to +15.4)	0.8
**Urine ACR**	Unadjusted	−0.1 (−0.6 to +0.5)	−0.5 (−0.7 to −0.2)	0.01
	Model 1	0 (−0.8 to +0.8)	−0.3 (−0.7 to 0)	0.11
	Model 2	−0.1(−0.8 to +0.6)	−0.4 (−0.8 to 0)	0.16

eGFR, estimated glomerular filtration rate, values provided in mL/min/1.73m^2^.

ACR, albumin‐to‐creatinine ratio, values provided in mg/g creatinine.

Mean estimates (95% confidence intervals) are provided.

Model 1: Covariates included age, sex, sickle cell genotype.

Model 2: Model 1 + *APOL1* kidney risk status.

Because clinical data are limited, current American Society of Hematology guidelines have conditional recommendations with low levels of certainty for the use of ACEi/ARB to treat sickle cell nephropathy.[2] In a longitudinal cohort of SCD patients, we demonstrate that there may be a benefit of ACEi/ARBs in reducing albuminuria independent of *APOL1* risk status. Nineteen percent (9/47) of SCD patients had to discontinue ACEi/ARB therapy due to adverse side effects potentially related to ACEi/ARB therapy. Discontinuation for adverse effects was more frequently observed in SCD patients with *APOL1* high risk. Our findings are consistent with the observed benefit of ACEi on kidney disease in the AASK cohort, although our study is limited by the small sample size. An additional limitation is the unavailability of other potential risk factors, such as socioeconomic and diabetes status or acute kidney injury events, which may affect kidney disease progression and should be evaluated in future prospective studies of ACEi/ARBs. Larger and longer follow‐up studies of ACEi/ARBs as well as new targeted therapies to treat sickle cell nephropathy, particularly in those that co‐inherit the high risk *APOL1* kidney risk variants, are urgently needed.

## CONFLICT OF INTEREST

The authors declare no relevant competing financial interests.

## AUTHOR CONTRIBUTIONS

Jin Han, Andrew Srisuwananukorn, Binal N. Shah, Santosh L. Saraf, Robert E. Molokie, James P. Lash, and Victor R. Gordeuk designed and performed the research, analyzed the data, and wrote the paper.
